# Estrogen receptors and the aging brain

**DOI:** 10.1042/EBC20200162

**Published:** 2021-11-26

**Authors:** Silvia Maioli, Karin Leander, Per Nilsson, Ivan Nalvarte

**Affiliations:** 1 Division of Neurogeriatrics, Department of Neurobiology, Care Sciences and Society, Center for Alzheimer Research, Karolinska Institutet, 171 77 Stockholm, Sweden; 2 Institute of Environmental Medicine, Karolinska Institutet, 171 77 Stockholm, Sweden; 3 Department of Biosciences and Nutrition, Karolinska Institutet, 141 57 Huddinge, Sweden

**Keywords:** Ageing, Alzheimer's disease, Estrogen receptor, Parkinson's disease, Sex differences, Sex hormones

## Abstract

The female sex hormone estrogen has been ascribed potent neuroprotective properties. It signals by binding and activating estrogen receptors that, depending on receptor subtype and upstream or downstream effectors, can mediate gene transcription and rapid non-genomic actions. In this way, estrogen receptors in the brain participate in modulating neural differentiation, proliferation, neuroinflammation, cholesterol metabolism, synaptic plasticity, and behavior. Circulating sex hormones decrease in the course of aging, more rapidly at menopause in women, and slower in men. This review will discuss what this drop entails in terms of modulating neuroprotection and resilience in the aging brain downstream of spatiotemporal estrogen receptor alpha (ERα) and beta (ERβ) signaling, as well as in terms of the sex differences observed in Alzheimer’s disease (AD) and Parkinson’s disease (PD). In addition, controversies related to ER expression in the brain will be discussed. Understanding the spatiotemporal signaling of sex hormones in the brain can lead to more personalized prevention strategies or therapies combating neurodegenerative diseases.

## Introduction

The female sex hormone estrogen (E2, 17β-estradiol) is essential for reproduction. It is mainly produced in the ovary after puberty and by the placenta at pregnancy (in the form of estriol), and its levels drop sharply at menopause. Like all sex hormones, E2 is a lipophilic steroid that can diffuse through the blood–brain barrier (BBB) to reach the brain parenchyma, where it regulates various processes linked to development, reproduction, emotion, and cognition. Additionally, E2 can be synthesized locally in the brain of men and women through cholesterol metabolism. Both E2 and the male sex hormone testosterone (an androgen) have been ascribed neuroprotective properties. This is perhaps most evident in ischemic brain injury, where sex hormones elicit neuroprotection that most likely contribute to the sex differences observed in the stroke incidence and survival rate at all adult ages [1,2]. At reproductive age, the incidence and mortality rates of stroke is higher in men than in women; however, after menopause more women are affected by stroke than men, suggesting a neuroprotective contribution of sex hormones [3]. The molecular and pathophysiological mechanisms behind such neuroprotective events are still largely unknown, but clearly both E2 and androgens modulate several neuroprotective pathways including immune response, neurogenesis, glial cell functions, and response to excitotoxicity [2,4–8]. In this review, we will discuss the expression and roles of the estrogen receptors in the healthy aging brain, as well as in neurodegenerative diseases such as AD and PD. We will conclude by discussing if there is a clinical potential in using hormonal therapies to combat neurodegeneration.

## Estrogen receptors

**Figure 1:**
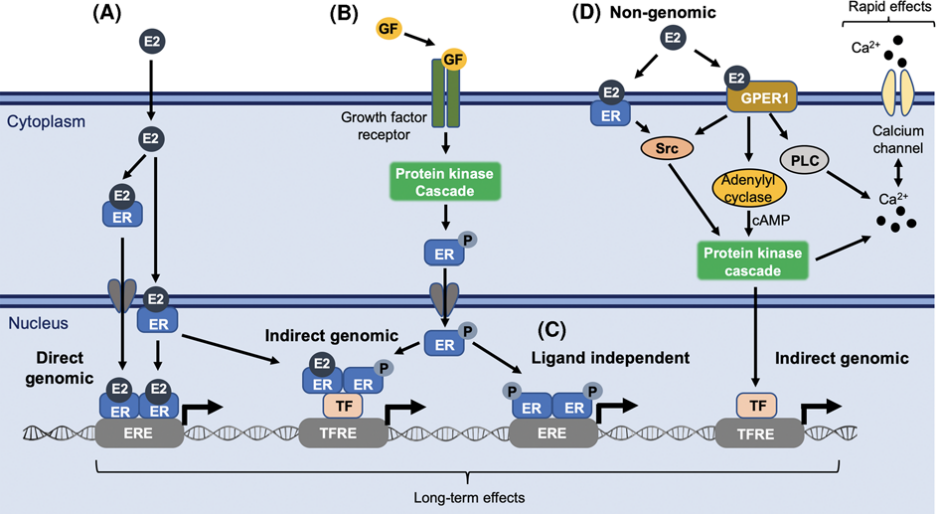
Schematic representation of ER signaling mechanisms (**A**) Classical genomic signaling. E2 binds ER in the cytoplasm or in the nucleus to mediate translocation, dimerization, and association of ERs to gene regulatory regions known as estrogen response elements (EREs). (**B**) Indirect genomic signaling (non-classical activation). Binding of growth factors (GFs) to growth factor receptors (GFRs) such as IGF-1R can activate PI3K and MAPK signaling pathways that in turn can phosphorylate ERs in the cytoplasm. The phosphorylated ERs translocate to the nucleus where they tether with other transcription factors on non-ERE sites (i.e. transcription factor response elements, TFREs) to modulate transcription of genes lacking EREs. The phosphorylated ER can also dimerize with ligand-bound ER to further modulate transcription or (**C**) bind to EREs in the absence of ligands to mediate gene-transcription. (**D**) Non-genomic signaling. E2 can bind to ERs in the cytoplasm (or at the plasma membrane) which directly interact with Src that modulates PI3K and MAPK signaling. In addition, E2 can bind the membrane bound GPER1 which also modulates kinase activations, either in a Src-mediated way or by stimulating adenylyl cyclase and cAMP production, which in turn can modulate different transcription factor activations, as well as phosphorylation of Ca^2+^ channels and influx of Ca^2+^. E2 binding to GPER1 can also activate the phospholipase C (PLC) pathway and mobilization of intracellular Ca^2+^ stores. This Ca^2+^ mobilization results in rapid non-genomic effects, adapting the cell to immediate responses, whereas the genomic signaling is slower, adapting the cell to long-term effects.

Three estrogen receptor subtypes exist; Estrogen receptor alpha (ERα), estrogen receptor beta (ERβ), and G-protein-coupled estrogen receptor 1 (GPER1). ERα and ERβ belong to the steroid activated nuclear receptor family of transcription factors. Upon E2 binding, they homo- or hetero-dimerize, recruit coactivators, and bind to estrogen response element (ERE) regulatory sites on the DNA to mediate transcription of genes involved in proliferation, differentiation, and survival (Figure 1). In addition to this classical genomic transcriptional activation, ERs may also tether to other transcription factors and act independently of E2, e.g., downstream of growth factor signaling (Figure 1) [9]. There are also several splice variants of the ERs, of which the ERβ splice variants have been studied most. These can dimerize with both ERα and ERβ and bind DNA, but lack, or have reduced, ligand binding activity, thereby modulating ER activity [9,10].

While the classical ERα and ERβ belong to the nuclear receptor family of transcription factors, GPER1 is a membrane-bound G-protein-coupled receptor without transcriptional activity and is suggested to mediate E2's rapid non-genomic actions linked to e.g., calcium flux and kinase activations and can cross-talk with the classical ERs (Figure 1) [11,12]. The exact molecular mechanisms behind this crosstalk and its overall contribution to E2 signaling is still largely unknown.

## Estrogen receptors in the brain

### Estrogen receptor expression in the brain

Both ERα and ERβ are overexpressed during neurodevelopment [13–15]. In rodents, ERs are responsible for masculinizing the brain during development since most of the androgens excreted by the developing testes from embryonic day 18 (E18) are converted into E2 by the aromatase enzyme (*Cyp19a1*) in the rodent brain [16]. In humans, however, the masculinization is mediated by brain androgen receptor (AR), although a role for ERs cannot be excluded [17,18]. Species differences in neuroendocrine control of adult brains also exist. For example, in the adult human brain, the highest expression of aromatase is found in thalamus, while rodents display the highest expression in amygdala [19]. There are also differences between humans and rodents in neuroendocrine control of gonadotropin-releasing hormone and in the distribution of ERs in different brain regions [20,21]. These important species differences should be taken into account when translating the influence of sex hormones between rodent and human brains.

Levels of ERs in the brain decrease after birth and their expression is confined to various brain regions. Studies mainly made on rodents show that, in the adult brain, both ERα and ERβ expression is overall low but with a wide distribution pattern [20,22–27]. However, this pattern of in particular ERβ, but also GPER1 and aromatase, is controversial due to issues related to antibody specificity to these proteins. This has resulted in confusion in the field of brain estrogen signaling. Several of the most widely used commercial ERβ antibodies have shown little or no specificity [28,29]. A recent study [28] could not detect any significant amounts of ERβ in the human brain using a validated antibody against human ERβ. In contrast, follow-up studies using the same antibody did detect ERβ in the mouse brain after optimizing antigen retrieval [30] or using frozen mouse brain sections [31]. In line with this, the expression of ERβ mRNA has previously been shown widely distributed in the rodent brain [32], which is similar to the expression pattern demonstrated by Merchenthaler and colleagues [23] using an in-house generated ERβ antibody. This latter study also detected different degree of overlap between ERβ and ERα immunoreactivity in different brain regions that correlated well with accumulation of ^125^I-labeled E2 in these regions [23]. In addition, a recent study using ERβ lineage tracing could detect significant amounts of ERβ in e.g., paraventricular nucleus (PVN), dorasal raphe nucles (DRN) and medial amygdala (MeA) of the mouse brain [33], while using multiple single cell sequencing data resources (i.e, Janelia portal https://hipposeq.janelia.org; Allen Brain Map https://portal.brain-map.org; Broad single cell portal https://singlecell.broadinstitute.org/single_cell) show varying but mostly low amounts of ER transcripts in both the rodent and human cortex and hippocampus. To date, the overall conclusion one can draw is that ERα is widely distributed at generally low levels in the rodent brain with highest expression in the amygdala and the hypothalamus, whereas ERβ expression is the main ER found in the cerebral cortex (although at low levels). ERβ is also expressed in the cerebellum, hippocampus, MeA, bed nucleus of the stria terminalis (BNST), PVN, anteroventral periventricular nucleus, substantia nigra, ventral tegmental area, and the DRN, which appears to be similar between rodents and humans [20,22–27]. Although there are no well-working commercial antibodies today for immunohistochemistry on GPER1 and aromatase, GPER1 is suggested to be expressed in most brain regions where either, or both, ERα or ERβ is expressed, including hippocampus, cerebral cortex, and the hypothalamus [11,34]. Only few studies have addressed aromatase mRNA distribution in the brain, showing that it is mainly expressed in the rat BNST and amygdala during development, and that its expression is much lower in adult brain areas, although this can be regulated by androgens and E2 [35,36].

### Estrogen receptors in neuroendocrine signaling

A recent systematic review assessing the contribution of sex hormones on activation of neural circuits controlling emotion and cognition as measured by functional magnetic resonance imaging (fMRI) showed different activation pattern during the follicular and luteal phases in normally cycling healthy women in several brain regions such as amygdala, anterior hippocampus, and several cortical regions [37]. Thus, circulating E2 and progesterone (P4, which will not be discussed in this review) can influence neural circuits controlling emotional and cognitive processing in humans.

However, the underlying mechanisms behind sex hormonal modulation of neural circuits are not well understood. Several studies have demonstrated that E2 can rapidly influence synaptic transmission and dendritic spine formation [38–40], as well as long term potentiation (LTP) [1,39], likely through non-genomic and genomic actions, respectively. Both the mitogen-activated protein kinase (MAPK) family and the phosphatidylinositol 3 kinase (PI3K) are modulated by E2 to rapidly promote dendritic spine formation [41–43], which at least in part can be regulated through extranuclear ERα and ERβ [44–47], and their interaction with cell surface receptors such as insulin growth factor 1 receptor (IGF-1R) [48,49]. In addition, E2 enhances rat hippocampal N-methyl-D-aspartate (NMDA) receptor-mediated currents [50,51], and the loss of ERβ resulted in aberrant gamma-aminobutyric acid (GABA)ergic interneuron function in the mouse motor cortex [6], suggesting that ERs intricately modulate synaptic neurotransmission, LTP, and behavior. Studies on knockout (KO) mouse models have shown that mice lacking ERα have aberrant sexual behavior, linked to hypothalamic actions of this receptor [52], and that these mice have unaffected spatial learning but impaired performance in the passive avoidance tests, suggesting a role for ERα in non-hippocampal dependent (or non-spatial dependent) memory [53]. On the other hand, ERβ KO mice have normal sexual behavior (although female ERβ KO mice have reduced fertility [54]), impaired spatial learning [55], increased locomotor activity [6], and increased anxiety behavior [56], but not impaired performance in the passive avoidance test [53], suggesting that ERα and ERβ have some degree of complementary functions in cognition. Furthermore, selective ERβ activation can modulate microglia-mediated neuroinflammation [57] and protects oligodendrocytes against myelination damage in a mouse model of multiple sclerosis [58]. Similar effects were also seen upon selective ERα activation, although this neuroprotection appears mediated explicitly via astrocytes [59]. Since ERs have important functions in neurodevelopment it is important to discriminate between their developmental effects and their functional effects in the adult brain, nevertheless, ERs function clearly beyond controlling sexual behavior and reproduction.

## Estrogen receptors and healthy ageing of the brain

In the course of healthy ageing a magnitude of brain-specific and systemic changes occur that have an impact on the resilience against adverse events. It has been demonstrated that the levels of both ERα and ERβ, found in synapses of CA1 neurons in the hippocampus of female rats, decrease with age [22,44,60]. If ER expression is linked to resilience in the brain, then this would imply that one level of neuroprotection is lost in the course of healthy ageing. However, since ERs function mainly as E2 binding proteins, the largest risk of their decreased *activity* in the brain would be when gonadal E2 levels drop. In women, a sharp drop in E2 levels occurs naturally at menopause. An increasing body of evidence suggest that the menopausal E2 drop in fact increases the vulnerability to adverse neurological events. This will be discussed in the following sections.

### Menopausal loss of gonadal estrogen—loss of resilience?

Women experience a drastic decrease in E2 levels at menopause. In contrast, aged men have a slow decrease in testosterone levels. Although testosterone has also been ascribed neuroprotective functions [61,62], it is still debated whether testosterone acts through brain AR or via its conversion to E2 by brain aromatase to elicit neuroprotective functions in humans [63]. Likely both are true to varying extents in different brain regions and under different pathological conditions, which implies that there may be sex differences between the female and male brain with respect to neuroprotection mediated by circulating sex hormones. Perhaps the most convincing evidence that links systemic loss of estrogen during menopause to neurological changes is the increased risk of depression associated with the menopausal transition (reviewed in [64]). This risk is significantly reduced in perimenopausal women treated with estrogen [65–67], lending further support to the hypothesis that systemic E2 levels do influence behavior. A similar reduction in risk has not been demonstrated in postmenopausal women who are older when initiating estrogen therapy [68], which is in line with the ‘critical time period hypothesis’ stating that estrogen treatment must be initiated early in relation to menopause onset to provide neuroprotection [69]. In addition, the type of hormone treatment (HT; E2 or in combination with progestins) and its duration may also have a bearing on the risk of depression, but knowledge of this is limited. Similarly, increasing evidence put forward a link between menopause and risk of subjective cognitive decline [70], which is often reported by menopausal women [71], and is associated with increased risk of developing AD later in life [72]. A recent systematic review demonstrated that the menopausal transition associates negatively with attention, and verbal and working memory [71]. Additionally, premenopausal women that have undergone oophorectomy have an increased risk of developing depression [73–75] and dementia later in life [74]. Similar findings have been found in premenopausal women that have undergone hysterectomy [76,77], suggesting that early removal of both E2 and P4 in premenopausal women affects mental health.

It would therefore be predicted that HT use in these women may not only alleviate symptoms of depression and cognitive decline but also prevent a possible excess risk of dementia. However, data on HT use as a modulator of present and future cognitive decline in postmenopausal women is controversial, owing to misinterpretations and inconsistencies, that in turn is a result of limited study population and not taking crucial biases into account, such as age, education, socioeconomic factors, and comorbid diseases. Further, factors related to HT type and its initiation in relation to menopause onset (e.g., the ‘critical time period hypothesis’) have not been sufficiently taken into account. Interestingly, HT treatment including both E2 and progestins appears to not elicit the same neuroprotective effects on cognitive decline as with E2 alone, in fact progestins may antagonize the protective effects of E2 [64,78,79].

## Brain cholesterol metabolism and estrogen receptors

In the brain, E2 can be synthesized *de novo*, mainly in neurons, in a process called neurosteroidogenesis [80]. Therefore, in order to present an overview on estrogen signaling in the brain, it is warranted to include a discussion on neurosteroidogenesis and how aberrant control of this process may modulate local E2 production.

Cholesterol acts as precursor of sex-hormone synthesis, and therefore an interplay between brain cholesterol metabolism and E2 signaling in the brain can be assumed. Cholesterol homeostasis is essential for healthy brain functioning, and cholesterol imbalance can trigger pathological processes underlying neurodegenerative diseases. Homeostasis of cholesterol is maintained by continuous effluxes of BBB-permeable oxysterols (sidechain-oxidized cholesterol metabolites) between the circulation and the brain [81–83]. 24-S-hydroxycholesterol (24SOH) and 27-hydroxycholesterol (27OH) are key players in maintaining such balance [82], by e.g., activating Liver X Receptors (LXRs) [84]. LXR signaling is involved in multiple pathways in the brain, including regulation of cholesterol synthesis and neurosteroidogenesis [85]. Upon activation, LXR can dimerize with retinoic acid receptor (RAR) [86] and promote transcription of genes involved in neurosteroidogenesis [87]. In the periphery, LXR has been found to decrease breast cancer growth through an ER-dependent mechanism [88] and AR down-regulates LXR signaling, leading to cholesterol accumulation in prostate cancer cells [89]. Such cross-talk between steroid hormone receptors, LXR signaling, and cholesterol metabolism can be suggested to occur in a variety of cellular contexts, including brain cells, and could differ between men and women. Conditions as hypercholesterolemia or BBB disruption may also affect this interplay. It is well known that cardiovascular risk factors such as hypercholesterolemia increase the risk of developing cognitive decline and AD, and the flux of 27OH into the brain has been considered as the missing link between hypercholesterolemia and AD [90–92]. 27OH levels are linked to cardiovascular disease and, importantly, it has been described as an endogenous selective estrogen receptor modulator (SERM) [93]. As a SERM, 27OH has different ER affinity and cell type selectivity [93], likely having different effects in periphery and brain. It still remains unclear how oxysterols affect E2 mediated neuroprotection, and to what extent hypercholesterolemia (with high 27OH) corresponds to AD risk. In addition to 27OH, the enzyme CYP46A1 converting brain cholesterol into excretable 24SOH has received increasing attention in relation to aging and neurodegeneration [94–96]. CYP46A1 overexpressing mice showed sex-specific differences in cognitive function, with females displaying positive memory effects, higher resilience against neural aging, as well as increased aromatase levels and increased ERβ signaling in hippocampus. On the opposite, males were found to develop anxiety-like behavior and memory deterioration, accompanied by high levels of dihydrotestosterone (DHT) [97]. In a memory clinic cohort, higher 24OH in the cerebrospinal fluid was found to correlate with lower neurofilament-light chain (a marker of axonal damage) and phospho-Tau only in women [97]. Taken together, these evidence highlights the need to further study brain cholesterol metabolism in relation to E2 signaling and the sex differences observed in neurodegenerative diseases.

## Estrogen receptors in neurodegeneration

Clear sex differences exist in the risk and pathophysiological progression of several neurodegenerative diseases. These sex differences are perhaps most apparent in AD and PD, and ERs have been ascribed important roles in the risk and progression of these neurodegenerative diseases.

### Estrogen receptors in Alzheimer’s disease

AD is the most common neurodegenerative disease, characterized by amyloid plaques of aggregated amyloid β peptide (Aβ), neurofibrillary tangles of hyperphosphorylated protein Tau, and neuroinflammation, leading to progressive neuronal atrophy, dementia, and death. Middle-aged women have a 2- to 3-fold increased risk compared with men in developing AD [98]. Although women on average live longer than men, it is clear that other factors also contribute to the increased AD risk in women, including sex hormones, genetic factors, and environment including geographic location [99–103]. In addition, women converts faster from mild cognitive impairment (MCI) to AD and present a more aggravated AD pathophysiology than men [104,105]. Women homozygous for APOE-ε4 run a greater risk of developing late-onset AD, while homozygous men run a greater risk of early-onset AD [106]. ApoE is involved in maintaining lipid/cholesterol metabolism in the brain, which in turn crosstalks with estrogen signaling (as discussed above). Taken together, this proposes a very complex interaction between sex hormones, cholesterol metabolism, genes and environment, which has restrained our understanding on the mechanisms behind the sex difference in AD. Nevertheless, an increasing body of evidence put forward E2 signaling through ERs as one important contributing factor to these sex differences.

E2 has been proposed to exhibit neuroprotective effects in experimental models of the AD brain [107,108]. However, the actual ER subtype contribution to the neuroprotection in AD is not well understood. Regarding ERα, studies have shown that this receptor is overexpressed in several neuronal nuclei of the basal forebrain, mammillary body, and hypothalamus in AD patients compared with sex- and age- matched healthy brains [109–112], while it is decreased in hippocampal neurons [113]. Some studies have also shown that ERα overexpression leads to increased Tau phosphorylation, while others show the opposite [114], and that ERα has protective effects on glutamate-mediated trauma in cultured rat neurons of female, but not male, origin [115]. The conflicting results have raised controversies to the actual contribution of ERα to neuroprotection in AD. However, it has been demonstrated that selective ERα activation in ovariectomized female transgenic AD mice (APP/PS1 and 3xTg AD mouse models) results in improved cognitive performance [116] and reduced Aβ levels in hippocampus, subiculum, and amygdala [117]. In addition, ERα gene polymorphisms in humans are associated with cognitive decline after menopause [118,119] and have been found in subsets of AD patients [120,121]. These data suggest that ERα has some role in AD risk and progression, although the molecular mechanisms and its overall neuroprotective contributions are still not clear.

In contrast, data on a neuroprotective role of ERβ in AD seems more consistent. A study on female AD brains detected decreased ERβ levels in the frontal cortex compared to age-matched controls, and they showed that ERβ was associated with mitochondria in these brains [122]. It appears that ligand binding to ERβ promotes its association with neuronal mitochondria and that loss of ERβ impairs the mitochondrial membrane potential and function [123–125]. These data could point toward neuronal exhaustion upon loss of ERβ. In line with this, ERβ KO mice display gradual progression of neurological deficits as they age, with significant loss of neuronal cell bodies throughout the brain in both sexes compared to wild-type littermates, in particular in substantia nigra [126]. Furthermore, studies using the 3xTg mouse model of AD demonstrated that selective ERβ activation is more effective than ERα activation in lowering Aβ levels in the frontal cortex and equally effective in the amygdala [117], and that long-term ERβ treatment improved spatial recognition memory, attenuated Aβ plaque formation, prolonged survival, and promoted physical health in these mice [127]. Although not well understood, molecular studies have suggested that, in addition to modulating mitochondrial health, ERβ can promote Aβ degradation through directly interacting with the autophagy machinery, thereby enhancing the clearance [128] and intracellular trafficking of Aβ [107], as well as by modulating microglia-mediated neuroinflammation [57] and interacting with cholesterol metabolism [97].

### Estrogen receptors in Parkinson’s disease

Parkinson’s disease is caused by age-related neurodegeneration in substantia nigra and/or loss of dopaminergic neurons, leading to motor dysfunction. Oppositely to AD, PD is more common in men than in women, and the age of PD onset is generally also earlier in men [129]. Men also display a more aggravated disease progression [103], which is mirrored in animal models of PD where neurotoxic hydroxydopamine administration results in increased motor deficits and nigrostriatal dopamine (DA) loss in male compared with female animals [130]. Additionally, the expression of genes in substantia nigra and striatum involved in PD pathogenesis and dopamine machinery is different between male and female PD brains [131–133].

E2 is proposed to have a modulatory effect on PD [134], and observational studies of postmenopausal women show a decreased risk of PD in women taking HT [135]. Of great interest is also a study linking HT to lower severity during the early phase of PD in women, prior to L-DOPA treatment [136], suggesting that E2 treatment could be useful in patients that already have developed PD. Similar disease-modifying effects of E2 has not been observed in AD. The beneficial effects of E2 may be associated with its facilitation of rapid DA release in the striatum and nucleus accumbens shown in rats [137,138], as well as with the neuroprotective properties of physiological E2 levels discussed above. Interestingly a role for testosterone in mediating neuroprotection in PD has not been convincingly demonstrated. In contrast with E2 treatment, administration of testosterone or DHT (which cannot be aromatized to E2) does not protect against neurotoxicity in the 1-methyl-4-phenyl-1,2,3,6-tetrahydropyridine (MPTP) model of PD but rather worsens pathology [134]. This implies that testosterone cannot be aromatized efficiently enough to exhibit neuroprotection in mouse models of PD, or that it has effects opposite of E2 in the brain. In line with this, aromatase knockout mice that lack the capacity of synthesizing E2 had increased pathology in the MPTP model compared to wild-type mice [139]. It was also shown that ERs are necessary for the neuroprotective effects of E2 in the MPTP model since, in contrast with 17β-estradiol (E2), 17α-estradiol, which cannot bind to ERs, does not protect against neurotoxicity [140]. Both ERα and ERβ are suggested to participate in this protection. In two studies, selective ERα activation was demonstrated to exhibit neuroprotection by preventing DA depletion and loss of dopamine transporter in the striatum [141] and preventing nigral dopaminergic cell death [142] in rodent models of PD. Similar effects could not be observed with a selective synthetic ERβ agonist in these studies. However, another study found that the endogenously produced 5-androstene-3β, 17β-diol, an ERβ agonist, protects against neuroinflammation in the rotenone-induced rat model of PD [143], suggesting that type of PD model and type of selective ER modulators may influence the neuroprotective readouts. Furthermore, a polymorphism in the human ERβ gene was more common in both sexes of early-onset PD patients compared with late-onset patients [144], whereas ERα polymorphisms appear to not be associated with PD [145].

## Estrogen receptors as clinical targets in neurodegeneration

As reviewed above, there is compelling evidence that E2 has neuroprotective functions, and that this is mediated by different ERs in different brain regions. It is therefore warranted to ask if E2 or a selective ER agonist treatment could be a clinical option in combating neurodegenerative diseases. Observational studies point towards a decreased risk of AD and PD in women taking at least menopausal estrogenic replacement therapy (ERT, without progestins) compared with never HT users [78,79,135]. However, the results and interpretations of these observational studies varies [135], which has impeded any clinical recommendations on HT use with regards to dementia risks. In addition, the use of HT is controversial since it can increase the risk of breast cancer, thromboembolism, and stroke. Lately, reassessment of old studies and addition of new knowledge has convincingly demonstrated that the benefits of HT can outweigh these risks. In particular, a large study demonstrated no increased risk of stroke exist when HT is initiated early after menopause [146]. Additionally, new data show that when adjusting for confounding factors, there is no increased breast cancer associated with HT, and even a lower risk in women only taking ERT [147,148]. Clearly, larger and more detailed epidemiological studies are needed to assess the contribution of menopausal E2 loss on PD, AD and non-AD dementia risk, and which individuals that may particularly benefit from estrogenic treatment or preventive interventions.

The preclinical data that exist on ERβ as a neuroprotective factor is very interesting from a therapeutic point of view. Since ERβ is mainly expressed in the brain, intestine and the gonads [23,28,30], it may be a promising target avoiding the side effects of systemic ERα activation. BBB permeable ERβ selective ligands are already in clinical trials for alleviating menopausal mood symptoms, and it would be interesting to explore their clinical potential in preventing neurodegeneration.

## Concluding remarks

Despite that E2 has been proposed as a neuroprotective factor in both AD and PD, it is interesting that women have an increased risk in developing AD, while men have an increased risk in being diagnosed with PD. How could loss of neuroprotective E2 at menopause increase the risk of AD but not of PD in women? And why does testosterone, which has also been ascribed neuroprotective roles, and which levels decrease much slower in aged men, not protect men from PD? Based on data described in this review, one can speculate that testosterone has, in the case of PD, the potential to be anti-neuroprotective, while in the case of AD be neuroprotective (possibly via its conversion to E2), and that brain-region specific differences exist in this neuroprotection. Thus, it is also tempting to speculate that, while testosterone is neuroprotective in regions affected in AD, such as cortex and hippocampus, it is not protective, or is not efficiently converted into E2, in PD affected regions, such as the midbrain, which in turn could be due to different spatial expression of aromatase between the two diseases. In addition, all aged women experience menopause, but why do only some develop AD? What other resilience factors cooperate with E2, and can local E2 production compensate postmenopausal drop of circulating sex hormones? Clearly, many factors interact with the risk of developing multifactorial AD and PD, including sex hormone levels, genes, comorbid diseases (such as cardiovascular disease), and the environment. In addition, interactions with neurodevelopmental processes should be taken into consideration, such as that microglia appears to display a more neuroprotective phenotype in females than males, and that this may originate from the surge in testosterone during the male brain masculinization [149,150]. Deciphering these interactions and the spatiotemporal involvement of sex hormone receptors will be tedious, warranting more accurate methods of detecting ERs and aromatase expression, and importantly requiring new integrative approaches combining preclinical, clinical, and detailed epidemiological data. Clearly, such integration can truly contribute to increase our knowledge on ER signaling in the ageing brain and whether this signaling impacts on resilience against neurodegenerative diseases, offering new clinical, more personalized, recommendations or treatment options for both men and women.

## Summary

- Understanding the spatiotemporal expression and signaling of estrogen receptors in the brain may offer new understanding to the normal and pathophysiological aging of the brain, and the sex differences observed in these processes.
- Although great achievements have been made on the topic of sex hormone signaling in the brain, a deeper understanding of the molecular mechanisms involving the different estrogen receptor subtypes and their neuroprotective responses in the course of human healthy and pathological ageing is largely missing.
- Integration of preclinical, clinical and epidemiological studies are needed to address the impact of estrogen receptor signaling in the ageing brain and their potential as therapeutic targets in neurodegeneration.

## Abbreviations

27OH: 27-hydroxycholesterol
24SOH: 24-S-hydroxycholesterol
DHT: dihydrotestosterone
ERT: estrogenic replacement therapy
LXR: Liver X Receptor
MCI: mild cognitive impairment
MPTP: 1-methyl-4-phenyl-1,2,3,6-tetrahydropyridine
RAR: retinoic acid receptor
SERM: selective estrogen receptor modulator
